# Changes in metabolic syndrome and its components and the risk of type 2 diabetes: a nationwide cohort study

**DOI:** 10.1038/s41598-020-59203-z

**Published:** 2020-02-11

**Authors:** Min-Kyung Lee, Kyungdo Han, Mee Kyoung Kim, Eun Sil Koh, Eun Sook Kim, Ga Eun Nam, Hyuk-Sang Kwon

**Affiliations:** 1 Division of Endocrinology and Metabolism, Department of Internal Medicine, Myongji Hospital, Hanyang University College of Medicine, Gyeonggi-do, Republic of Korea; 2 Department of Medical Statistics, College of Medicine, The Catholic University of Korea, Seoul, Republic of Korea; 3 Division of Endocrinology and Metabolism, Department of Internal Medicine, Yeouido St. Mary’s Hospital, College of Medicine, The Catholic University of Korea, Seoul, Republic of Korea; 4 Division of Nephrology, Department of Internal Medicine, Yeouido St. Mary’s Hospital, College of Medicine, The Catholic University of Korea, Seoul, Republic of Korea; 5 Division of Endocrinology and Metabolism, Department of Internal Medicine, Incheon St. Mary’s Hospital, College of Medicine, The Catholic University of Korea, Seoul, Republic of Korea; 6 Department of Family Medicine, Korea University Anam Hospital, Korea University College of Medicine, Seoul, Republic of Korea

**Keywords:** Diabetes, Metabolic syndrome

## Abstract

We investigated the relationship of changes in Metabolic syndrome (MetS) and its components with the risk of type 2 diabetes (T2D) in South Korea. Records of 10,806,716 adults aged ≥ 20 years without a history of T2D between 2009 and 2015 were retrieved from database of the South Korean National Health Insurance Service and analyzed. Changes in metabolic components were monitored over a two-year period with follow-up occurring at an average of 4.087 years. During the follow-up period, 848,859 individuals were diagnosed with T2D. The risk of diabetes was lowered with a decrease in the number of MetS components at baseline and the second visit (*p* for trend <0.0001). Multivariable-adjusted HRs for incident diabetes were 0.645 among individuals with reduced number of MetS components, 0.54 for those with improvement in elevated fasting glucose, 0.735 for those with improvement in elevated triglycerides, 0.746 for those with improvement in elevated blood pressure, 0.763 for those with improvement in reduced HDL-cholesterol, and 0.92 for those with improvement in abdominal obesity compared with those manifesting them at both time points. In conclusion, changes in metabolic syndrome and its components were significantly associated with the development of T2D. Improvement in MetS and its components attenuated the risk of diabetes.

## Introduction

Type 2 diabetes (T2D) is a chronic disease resulting from a complex interaction between heredity and environment, along with other risk factors^1^. Insulin resistance, obesity, and behavioral factors such as physical activity, diet, smoking, alcohol consumption, and body weight are important risk factors for T2D^2^. Increasing incidence of diabetes and related complications imposes a heavy health burden^3^. Thus, investigations should focus on effective interventions and preventive measures for the disease.

Metabolic syndrome (MetS) was conceptualized based on a constellation of risk factors, such as elevated fasting plasma glucose (FPG), atherogenic dyslipidemia, elevated blood pressure, and abdominal obesity, in individuals susceptible to cardiovascular disease (CVD) and T2D^4^. Guidelines for clinical and epidemiological management of MetS have been developed using readily available clinical variables^5^. A number of studies have demonstrated a strong association of MetS and its components with the risk of incident T2D^6–8^. Thus, clinical implications of MetS should be focused on multifactorial interventions to reduce the risk of T2D^9^. All individuals deserve long-term management of MetS and follow-up in the clinical setting^10^. However, cohort studies that correlate the development of T2D with changes in MetS and its components are lacking. Thus, the objective of this nationwide population-based cohort study was to investigate association of changes in MetS and its components with risk of T2D in South Korea.

## Methods

### Study subjects

We used the database provided by the National Health Insurance Service (NHIS), the single insurer managed by the South Korean government^11^. The NHIS public database contains data pertaining to health care utilization, health screening, socio-demographic variables, and mortality of the whole population (more than 51 million) of South Korea including information obtained from medical treatment and health screening records. Individuals enrolled in the NHIS are recommended to undergo standardized health examinations every two years. Disease diagnoses in the database are classified according to the International Classification of Disease-Tenth Revision-Clinical Modification (*ICD-10-CM*) codes. This study was approved by the NHIS inquiry commission, and the Institutional Review Board of The Catholic University of Korea (No. SC18ZESIO047).

**Figure 1:**
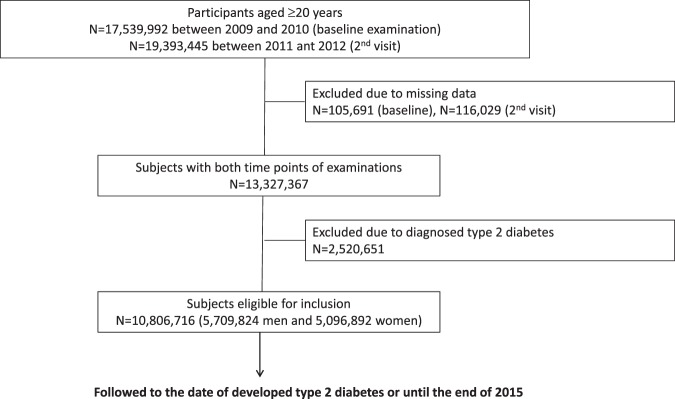
Flow chart of patient enrollment. Patients were followed by the development of type 2 diabetes or until the end of 2015.

From this cohort, the subjects aged ≥20 years who had participated in health examinations between 2009 and 2010 and were reexamined in 2011 and 2012 were selected. 17,539,992 individuals received health examinations at baseline and 19,393,445 individuals were examined two years later. We then excluded 105,691 individuals at baseline examination and an additional 116,029 at the second visit who had missing data involving at least one variable. Among 13,327,367 individuals with both time points of health examinations, we excluded 2,520,651 who had been diagnosed with T2D. Finally, a total of 10,806,716 individuals (5,709,824 men and 5,096,892 women) were included in this study. They were followed up to monitor the development of T2D or until the end of 2015 with a median follow-up duration of 4.087 years (Fig. 1).

### Diagnosis of type 2 diabetes

The NHIS database (DB) represents the entire Korean population^12^. Among the total datasets of the NHIS DB, we used qualifications, claims, the health check-up DB, and death information. Due to the inevitable limitations of the claim DB, T2D was defined operationally^13^. T2D from the claim DB was categorized based on the ICD-10 code of T2D (E11–E14) as principal diagnosis or up to a fourth additional diagnosis and at least one antidiabetic drug prescribed in a given year. T2D from health check-up DB was defined based on FBG (undiagnosed diabetes) or the ICD-10 code of T2D with a claim for antidiabetic medication. To validate the information accuracy, an expert committee from the Korean Diabetes Association reviewed the dataset regularly. The committee decided on the suitability of the dataset and reviewed the results of the analysis.

### Definition of metabolic syndrome

MetS was defined by a combination of abdominal obesity, impaired fasting glucose, atherogenic dyslipidemia, and elevated blood pressure. Revised NCEP ATP III criteria^10,14^ require at least three of the following components: (1) abdominal obesity (waist circumference [WC] ≥90 cm for men, or ≥85 cm for women)^15^; (2) triglycerides ≥150 mg/dL, and/or drug treatment for elevated triglycerides; (3) high-density lipoprotein (HDL)-cholesterol <40 mg/dL for men, or <50 mg/dL for women; (4) systolic blood pressure (BP) ≥130/85 mmHg or antihypertensive medication treatment, and/or a history of hypertension; and (5) FPG ≥100 mg/dL, and/or treatment with medications for T2D.

### Measurement of covariates

Height, weight, WC, and BP was measured during regular medical check-ups in accordance of the heath checkups implementation standard^16^. Brachial BP was measured after 5 minutes of rest in a sitting position. The BP measurement was repeated if the first measurement exceeded 120/80 mm Hg. Body mass index (BMI) was calculated as weight in kilograms divided by height in meters squared (kg/m^2^). Serum samples for measuring FPG, total cholesterol (TC), HDL-cholesterol, and triglyceride levels were obtained after overnight fasting before each examination. Detailed histories of smoking status, alcohol consumption, physical activity, and past medical history were obtained using a self-administered questionnaire. Based on smoking status, subjects were classified into non-smokers, ex-smokers, or current smokers. Individuals who consumed ≥30 g of alcohol per day were defined as heavy alcohol drinkers. Physical activity was categorized by the frequency of ≥20 minutes of strenuous exercise. Hospitals conducting health examinations were certified by the NHIS and regularly monitored for quality control.

### Statistical analyses

Baseline characteristics of individuals according to T2D status were compared using Student’s *t*-test and Pearson’s Chi-square test for continuous and categorical variables, respectively. We determined the association between changes in MetS components over a two-year period and the incidence of T2D during four years of follow-up using Cox proportional hazards regression analysis. Hazard ratios (HRs) and 95% confidence intervals (CIs) were calculated. HRs were adjusted for sociodemographic characteristics. Model 1 was adjusted for age and sex. Model 2 was adjusted for age, sex, alcohol consumption, smoking status, physical activity, and BMI. All statistical tests were two-sided, and *p* < 0.05 was considered significant. All analyses were performed using the Statistical Analysis System statistical software package, version 9.4 (SAS Institute Inc., Cary, NC, USA).

### Ethics approval and consent to participate

This study was approved by the NHIS inquiry commission and adhered to the tenets of the Declaration of Helsinki for biomedical research. Informed consent was not required because the national insurance claim data were deidentification for the analysis. This study was also approved by the Institutional Review Board of The Catholic University of Korea (No. SC18ZESIO047).

## Results

### General baseline characteristics

During a median follow-up of 4.087 years, a total of 848,859 individuals were diagnosed with T2D. Table 1 presents baseline demographics of the study population according to new-onset T2D status. Individuals who developed T2D were more likely to be older and males with higher BMI, WC, TC, BP, and FPG values than those without T2D. They also had higher rates of MetS (41.15% vs. 18.65%), abdominal obesity (28.31% vs. 15.63%), elevated triglycerides (45.51% vs. 29.48%), reduced HDL-cholesterol (37.36% vs. 23.15%), elevated BP (58% vs. 37.08%), and elevated FPG (37.94% vs. 22.28%).

**Table 1:** Baseline Clinical Demographics of Participants Diagnosed with Type 2 Diabetes During Follow-up.

	New-onset type 2 diabetes		*p-*value
	No	Yes	
Participants, n	9888223	848859	
Age (years)	47.19 ± 13.06	57.6 ± 12.3	<0.0001
Male (%)	423272 (49.86)	5286552 (53.09)	<0.0001
Body mass index (kg/m²)	23.54 ± 3.1	24.76 ± 3.26	<0.0001
Waist circumference (cm)	79.47 ± 8.89	83.82 ± 8.73	<0.0001
Total cholesterol (mg/dL)	195.44 ± 35.22	194.33 ± 39.71	<0.0001
Triglycerides (mg/dL)*,	103 (72–154)	122 (85–179)	<0.0001
HDL cholesterol (mg/dL)	55.81 ± 14.56	53.64 ± 14.41	<0.0001
Systolic blood pressure (mmHg)	120.91 ± 14.28	126.82 ± 15.2	<0.0001
Diastolic blood pressure (mmHg)	75.6 ± 9.76	77.77 ± 9.93	<0.0001
Fasting plasma glucose (mmol/l)	92.31 ± 10.61	100.9 ± 13.42	<0.0001
Metabolic syndrome (%)	1857347 (18.65)	349302 (41.15)	<0.0001
Elevated fasting glucose (%)	2218827 (22.28)	322049 (37.94)	<0.0001
Elevated triglycerides (%)	2935369 (29.48)	386278 (45.51)	<0.0001
Reduced HDL-cholesterol (%)	2305559 (23.15)	317150 (37.36)	<0.0001
Elevated blood pressure (%)	3692653 (37.08)	492302 (58)	<0.0001
Abdominal obesity (%)	1556385 (15.63)	240280 (28.31)	<0.0001
Current smoker (%)	269527 (19.55)	2308698 (23.4)	<0.0001
Alcohol drinker (%)	582198 (5.86)	51101 (6.04)	<0.0001
Regular physical activity (%)	5513578 (55.8)	702578 (48.85)	<0.0001

Data are presented as mean ± standard deviation (SD) or proportions (%).

*Geometric mean.

### Changes in the number of MetS components and the risk of T2D

Table 2 shows associations between changes in number of MetS components and risk of T2D. For individuals with the same number of MetS components at baseline examination, HR for incident T2D was lower for lower numbers of MetS components at the second visit than at baseline, and vice versa, it was higher as the number increased. After adjusting for age, sex, alcohol drinking, smoking status, exercise, and BMI, HR for incident T2D was 0.51 (95% CI: 0.497–0.522) for individuals with four to five MetS components at baseline and zero to one component at the second time point. It was 0.268 (95% CI: 0.266–0.271) for those with zero to one component at baseline and zero to one component at the second visit compared to those with four to five components at both time points (Table 2).

**Table 2:** Hazard Ratios (95% CI) for Incident Type 2 Diabetes According to Changes in Number of Metabolic Syndrome Components from Baseline to the Second Visit.

No. of components		No.	Cases, n	Follow-up duration (person-years)	Incidence rate (per 1,000 person-years)	HR (95% CI)	
Baseline	2^nd^ visit					MODEL1	MODEL2
4,5	4,5	607370	323806	2601446.3	124.472	1 (ref.)	1 (ref.)
	3	272829	87487	1225757.06	71.374	0.727 (0.718, 0.735)	0.744 (0.735, 0.752)
	2	147504	35700	677738.11	52.675	0.598 (0.589, 0.608)	0.617 (0.607, 0.626)
	0,1	67926	10613	319037.6	33.266	0.482 (0.47, 0.494)	0.51 (0.497, 0.522)
3	4,5	336591	114105	1493535.62	76.399	0.851 (0.842, 0.86)	0.864 (0.855, 0.873)
	3	488771	116144	2239703.01	51.857	0.637 (0.63, 0.643)	0.66 (0.653, 0.667)
	2	404229	67614	1897444.11	35.634	0.511 (0.505, 0.517)	0.534 (0.528, 0.54)
	0,1	283606	30233	1358389.06	22.257	0.396 (0.39, 0.402)	0.424 (0.418, 0.431)
2	4,5	210854	55861	950753.22	58.754	0.761 (0.751, 0.771)	0.777 (0.768, 0.787)
	3	464831	79523	2168358.98	36.674	0.56 (0.554, 0.566)	0.583 (0.577, 0.59)
	2	779499	103786	3700729.29	28.045	0.44 (0.436, 0.445)	0.467 (0.462, 0.471)
	0,1	910113	69895	4415757.5	15.829	0.33 (0.326, 0.333)	0.359 (0.355, 0.363)
0,1	4,5	107365	20352	491251.63	41.429	0.673 (0.661, 0.684)	0.701 (0.689, 0.713)
	3	376749	44911	1781363.82	25.212	0.478 (0.472, 0.484)	0.507 (0.501, 0.513)
	2	1033834	82456	4986623.56	16.535	0.36 (0.356, 0.363)	0.388 (0.384, 0.392)
	0,1	4835414	196776	23793248.67	8.27	0.239 (0.237, 0.241)	0.268 (0.266, 0.271)

Model 1 was adjusted for age and sex. Model 2 was adjusted for age, sex, alcohol consumption, smoking status, physical activity, and BMI.

**Figure 2:**
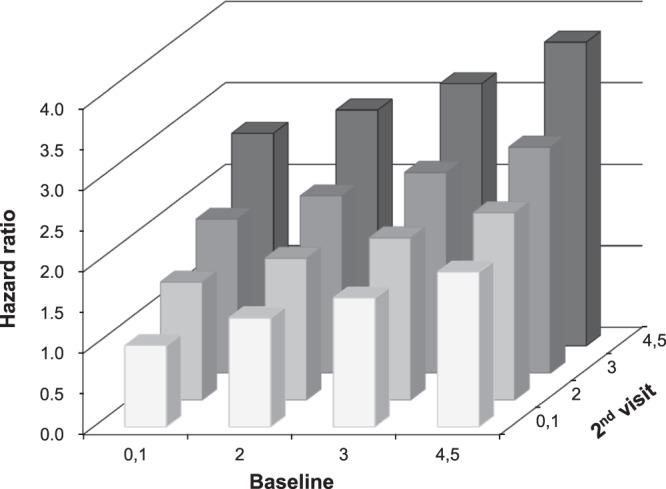
Hazard ratios (95% CI) for incident diabetes according to changes in number of metabolic syndrome components. The risk of diabetes was increased with the number of metabolic syndrome components at baseline examination and the second visit (*p* for trend < 0.0001).

HRs for incident T2D decreased as the number of MetS components at baseline decreased. Likewise, the lower the number at the second visit, the lower the risk of incident T2D (both *p* for trend <0.0001). The risk of T2D increased with the number of MetS components at both time points (*p* for trend* < *0.0001, Fig. 2).

Multivariable hazard ratios were adjusted for age, sex, alcohol drinking, smoking status, physical activity, and BMI.

### Changes in MetS and its components and the risk of T2D

Table 3 shows associations of changes in MetS and its components with the risk of T2D. The incidence rate of incident T2D was the highest in individuals with MetS or its components at both time points. It was the lowest in those without MetS or its components at both time points. Individuals with MetS or its components at baseline but without them at the second visit showed a decreased incidence of T2D (Table 3). HR for incident T2D was the lowest in individuals without MetS or its components at both time points. It was higher in those without MetS or its components at baseline and with them at the second visit than those with them at baseline but without them at the second visit.

**Table 3:** Multivariable-adjusted Hazard Ratios for Type 2 Diabetes According to Changes in Metabolic Syndrome and Its Components.

MetS and its components	Status		No.	Cases, n	Follow-up duration	Incidence rate	HR (95% CI)	
	Baseline	2^nd^ visit					MODEL1	MODEL2
Metabolic syndrome	Yes	Yes	1348002	258335	4858948	53.1669	1(ref.)	1(ref.)
	Yes	No	858647	90967	3278848	27.7436	0.609 (0.605, 0.614)	0.645 (0.64, 0.65)
	No	Yes	1105518	134303	4152818	32.3402	0.727 (0.722, 0.732)	0.76 (0.755, 0.765)
	No	No	7494549	365254	29490297	12.3856	0.374 (0.372, 0.376)	0.43 (0.427, 0.432)
Elevated fasting glucose	Yes	Yes	6808054	373209	26699935	47.674	1(ref.)	1(ref.)
	Yes	No	1365996	118208	5281524	22.3814	0.52 (0.516, 0.523)	0.54 (0.537, 0.544)
	No	Yes	1457786	153601	5523729	27.8075	0.642 (0.638, 0.646)	0.656 (0.651, 0.66)
	No	No	6808054	373209	26699935	13.9779	0.375 (0.373, 0.377)	0.404 (0.401, 0.406)
Elevated triglycerides	Yes	Yes	6144925	334902	24034506	34.7754	1(ref.)	1(ref.)
	Yes	No	1119128	98029	4315085	22.7177	0.689 (0.684, 0.694)	0.735 (0.73, 0.741)
	No	Yes	1340144	127679	5142447	24.8285	0.777 (0.771, 0.782)	0.815 (0.81, 0.821)
	No	No	6144925	334902	24034506	13.9342	0.51 (0.507, 0.512)	0.588 (0.585, 0.591)
Reduced HDL-cholesterol	Yes	Yes	6950041	394884	27259745	38.6107	1(ref.)	1(ref.)
	Yes	No	1072221	95340	4122993	23.124	0.725 (0.72, 0.731)	0.763 (0.757, 0.769)
	No	Yes	1233966	136825	4653389	29.4033	0.881 (0.875, 0.887)	0.91 (0.904, 0.917)
	No	No	6950041	394884	27259745	14.486	0.555 (0.552, 0.558)	0.621 (0.617, 0.624)
Elevated blood pressure	Yes	Yes	5211154	244220	20463164	36.4587	1(ref.)	1(ref.)
	Yes	No	1206373	86152	4717310	18.263	0.695 (0.69, 0.7)	0.746 (0.741, 0.752)
	No	Yes	1410607	112337	5460447	20.5729	0.76 (0.755, 0.765)	0.799 (0.794, 0.804)
	No	No	5211154	244220	20463164	11.9346	0.553 (0.55, 0.556)	0.638 (0.635, 0.642)
Elevated waist circumference	Yes	Yes	1157171	167263	4307384	38.8317	1(ref.)	1(ref.)
	Yes	No	768305	81784	2911413	30.2415	0.766 (0.759, 0.772)	0.92 (0.912, 0.928)
	No	Yes	639494	73017	2414465	28.0908	0.786 (0.78, 0.793)	0.904 (0.896, 0.912)
	No	No	8241746	526795	32147649	16.3867	0.553 (0.55, 0.556)	0.794 (0.788, 0.8)

Model 1 was adjusted for age and sex. Model 2 was adjusted for age, sex, alcohol consumption, smoking status, physical activity, and BMI.

**Figure 3:**
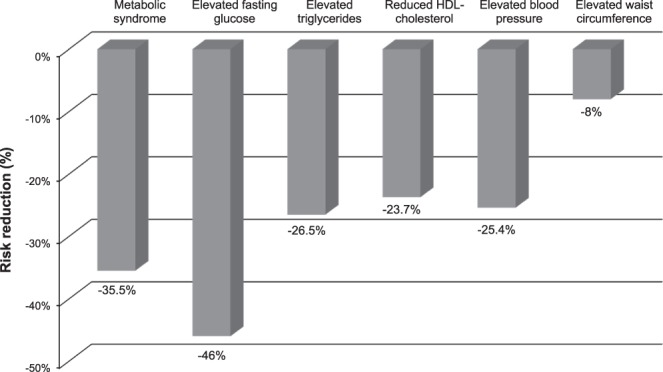
Risk reduction based on improvement in metabolic syndrome and its components.

Figure 3 describes risk reduction of diabetes according to improvement in MetS and its individual components. Multivariable-adjusted HR for incident T2D was 0.645 (95% CI: 0.64–0.65) in individuals with reduced number of MetS components compared to those with MetS at both time points. HRs for incident T2D were 0.54 (95% CI: 0.537–0.544) for those with improvement in elevated fasting glucose, 0.735 (95% CI: 0.73–0.741) for those with improvement in elevated triglycerides, 0.763 (95% CI: 0.757–0.769) for those with improvement in reduced HDL-cholesterol, 0.746 (95% CI: 0.741–0.752) for those with improvement in elevated blood pressure, and 0.92 (95% CI: 0.912–0.928) for those with improvement in elevated waist circumference compared to those with each component of MetS at both time points (Fig. 3).

## Discussion

In this prospective study of 10,806,716 adults, we found that changes in MetS and its components were associated with the risk of incident T2D. The risk of T2D was significantly lowered with a decrease in the number of MetS components at both time points regardless of the type of MetS component. In addition, improving MetS and its components reduced the risk of T2D.

The incidence of MetS has increased markedly in Korea^17^. Numerous epidemiological studies have demonstrated that MetS and its components can predict the development of T2D^18^. In the current longitudinal study, we found that improvement in MetS and its components was associated with a reduced risk of T2D. After adjusting for confounding factors such as age, sex, alcohol consumption, smoking status, exercise, and BMI, we found a 36% decrease in diabetes risk among individuals with reduced number of MetS components at the second visit, a 46% decrease with improvement in elevated FPG, a 27% decrease with improvement in elevated triglycerides, a 25% decrease with improvement in elevated blood pressure, a 24% decrease with improvement in reduced HDL-cholesterol, and a 8% decrease with improvement in abdominal obesity. Although it was not feasible to compare relative risks of changes in MetS and its components directly, the risk of diabetes was further reduced in individuals with improvement of elevated FPG than in those with improvement in MetS. Among MetS components, improvement in elevated FPG greatly reduced the risk of T2D.

Elevated FPG level has been comprehensively investigated as a core component in models for risk assessment of T2D^19,20^. Analysis of the Tehran Lipid and Glucose Study showed a positive association between changes in FPG levels and the incidence of T2D^21^. As expected, our results indicated that changes in FPG levels were most strongly associated with the development of T2D. It is well known that elevated FPG is a predictor of progression to diabetes^22^. Some studies have reported that individuals with MetS manifest a higher risk of developing diabetes than those with elevated FPG^23^ whereas other studies have indicated that elevated FPG is more effective than MetS for predicting incident diabetes^24^. In addition, it has been suggested that MetS is less strongly associated with the risk of T2D in Asian populations than in Western populations^25^. This discrepancy may result from differences in the degree of insulin resistance of these populations^26^. The current study showed that elevated FPG was a more potent component than MetS for the prevention of T2D.

In this study, we found that all MetS components were potentially related to the risk of incident T2D. Elevated triglyceride and reduced HDL-cholesterol are common dyslipidemic features accompanying T2D^27^. Some studies have shown that changes in fasting triglyceride levels are predictive of T2D, independent of traditional risk factors^28^. Reduced HDL-cholesterol is associated with an increased risk of T2D^29^. An increase in triglyceride level, particularly when it is accompanied by low HDL-cholesterol, has consistently been shown to be a surrogate marker of insulin resistance^30^ which is a strong predisposing condition for T2D. Elevated BP has been identified as a risk factor for T2D^31^. A strong association between abdominal obesity and the development of T2D has been recognized^32^. Interestingly, after adjusting for traditional risk factors such as BMI, we found that change in abdominal obesity remained a significant predictor of T2D. Indeed, when we analyzed the contribution of each MetS component, our findings showed an independent correlation of each component with diabetes risk. Thus, improving each component of MetS can reduce the risk of diabetes.

Lifestyle factors such as diet and physical activity can strongly influence metabolic parameters such as FPG, triglycerides, HDL-cholesterol, BP, and waist circumference^33^. Clinical trials have shown that lifestyle modifications can substantially reduce the risk of developing diabetes^34^. Smoking and excessive alcohol consumption are important risk factors for the development of MetS and its components^35,36^. The level of metabolic parameters at a single time point may inaccurately reflect the long-term risk of T2D. Therefore, we investigated the association of T2D with changes in metabolic parameters at two different time points in the current study. The effect of controlling MetS and its components on the development of T2D was determined after adjusting for age, sex, alcohol consumption, smoking status, physical activity, and BMI. The present longitudinal study indicated that changes in MetS and its components remained independent determinants of diabetes risk even after adjusting for lifestyle factors.

Strengths of the present study include its large sample size and a prospective cohort study design which ensured a thorough follow-up. In addition, the level of metabolic parameters at a single time point may inaccurately reflect the long-term risk of developing diabetes. We demonstrate a significant association of changes in MetS and its components with the risk of T2D. Our findings could be a cornerstone for other studies to explore the impact of changes in MetS on the risk for T2D-related outcomes. However, this study has several limitations. First, the four-year follow-up period was relatively short. Results might have been more significant with a longer follow-up. Second, diagnostic criteria for MetS vary between populations belonging to different ethnicities. Results may differ according to other diagnostic criteria. Nevertheless, the modified criteria of NCEP ATP III have been more strongly associated with T2D than Internal Diabetes Federation-defined MetS in Korean adults^37^. Third, the incidence of T2D might have been underestimated because individuals with undiagnosed T2D or those who failed to visit a hospital during the study period were excluded from this study. Fourth, this analysis relied on the NHIS DB. Basic laboratory tests, including fasting glucose, are performed in this health check-up program. Therefore, we could not obtain other laboratory values, such as glycosylated hemoglobin levels. In addition, these data do not include uninsured events because the data are basically made for claim purposes and only represent the services covered by the NHIS. For these reasons, discrepancies between the actual diagnosis and the NHIS DB might be possible. However, the operational definitions of T2D, which have been used in several studies, are appropriately validated to minimize inconsistent and inaccurate results. The NHIS DB represents the entire Korean population; therefore, it can be used in a population-based nationwide study for T2DM in Korea. Finally, although we adjusted for potential confounding factors, lifestyle factors that were unaccounted for in this study might have mediated changes in MetS. Further high-quality studies are needed to corroborate these results.

## Conclusions

In this large-scale cohort study, we found that longitudinal changes in MetS and its components were significantly associated with the development of T2D. Improvement in MetS and its components reduced the risk of diabetes, independent of lifestyle factors. Changes in each of MetS components could predict the development of T2D. In Korea, improvement in elevated FPG was more strongly related to a reduced risk of T2D than improvement in MetS. Although our present study could not establish causal relationships, the modifying effect of changes in MetS and its components on diabetes risk may provide new insights into strategies to prevent T2D.

## Data Availability

The data that support the findings of this study are available from the National Health Insurance Service but restrictions apply to the availability of these data, which were used under license for the current study, and so are not publicly available. Access to the dataset can be obtained through the Health Insurance Data Service home page (http://nhiss.nhis.or.kr).
